# Clinical and patient-reported outcome after patient-specific 3D printer-assisted cranioplasty

**DOI:** 10.1007/s10143-023-02000-9

**Published:** 2023-04-19

**Authors:** Florian Ebel, Stephan Schön, Neha Sharma, Raphael Guzman, Luigi Mariani, Florian M. Thieringer, Jehuda Soleman

**Affiliations:** 1 Department of Neurosurgery, University Hospital of Basel, Basel, Switzerland; 2 Department of Oral & Cranio-Maxillo-Facial Surgery and 3D Print Lab, University Hospital of Basel, Basel, Switzerland; 3 Medical Additive Manufacturing Research Group (Swiss MAM/Smart Implants), Department of Biomedical Engineering, University of Basel, Allschwil, Switzerland; 4 Department of Pediatric Neurosurgery, University Children’s Hospital of Basel, Basel, Switzerland; 5 Faculty of Medicine, University of Basel, Basel, Switzerland

**Keywords:** Patient-specific implant, 3D printing, Cranioplasty, Cosmetic, Computer-assisted surgery, Bone cements, Point-of-care implant

## Abstract

Various cranioplasty techniques exist for the reconstruction of cranial bone defects. Patient-specific implants can be produced in-house using a recently developed 3D printer-assisted cranioplasty technique. However, the resulting cosmetic outcomes from the patient’s perspective are underreported. With our case series, we aim to present the clinical outcome, morbidity rate, patient-reported cosmetic results, and cost-effectiveness of patient-specific3D printer-assisted cranioplasty technique. This is a consecutive retrospective case series of adult patients undergoing cranioplasty using the patient-specific 3D printer-assisted technique. As primary endpoint, the functional outcome based on modified Rankin scale (mRS) at discharge and follow-up was assessed. A prospective telephone survey was conducted to collect and provide patient-reported outcomes. Thirty-one patients underwent patient-specific 3D printer-assisted cranioplasty, mostly to reconstruct frontotemporoparietal (61.3%) and frontotemporal defects with orbital involvement (19.4%). Good functional outcome (mRS ≤ 2) at discharge and during the last follow-up was achieved in 54.8% (*n* = 17) and 58.1% (*n* = 18) patients. Overall, the rate of clinically relevant surgery-related complications was 35.5% (*n* = 11). Postoperative epidural hematoma/collection (16.1%) and infections (12.9%) were the most frequent complications. Permanent morbidity occurred in one patient (3.2%) with postoperative acute ipsilateral vision loss after frontotemporal cranioplasty with orbital involvement. No surgery-related mortality occurred. The mean patient-reported cosmetic satisfaction score was 7.8 ± 1.5, with 80% of patients reporting satisfying or very satisfying cosmetic results. No significant differences were seen between the different defect localization regarding the cosmetic outcome. The mean manufacturing costs of a patient-specific 3D printer-assisted implant ranged from 748 to 1129 USD. Based on our case series, patient-specific 3D printer-assisted cranioplasty is cost-effective and leads to satisfying cosmetic results, especially in large defects and/or defects with complex geometry.

**Supplementary Information:**

The online version contains supplementary material available at 10.1007/s10143-023-02000-9.

## Introduction

For various reasons, performing cranioplasty after cranial surgery may be necessary. So far, if the autologous bone is unavailable, bone reconstruction has mainly been achieved by using freehand techniques with polymethyl-methacrylate (PMMA). Alternatively, expensive industrially manufactured patient-specific implants (PSIs) made of various materials such as titanium or polyetheretherketone (PEEK) can be used [1]. Due to the affordable procurement costs, three-dimensional (3D) printers have recently been used for the in-house fabrication of patient-specific bone implants [1, 2]. According to the international consensus statement published in 2021 on posttraumatic cranioplasty, more research should be done to reduce the cost of patient-specific cranioplasty and improve cosmetic outcomes [3]. The literature on the patient-reported cosmetic outcome of patient-specific 3D printer-assisted cranioplasty is scarce [4].

This study aims to show the different applications of patient-specific 3D printer-assisted cranioplasty, the clinical outcome, morbidity rate, patient-reported cosmetic outcomes, and the associated costs.

## Methods

**Fig. 1:**
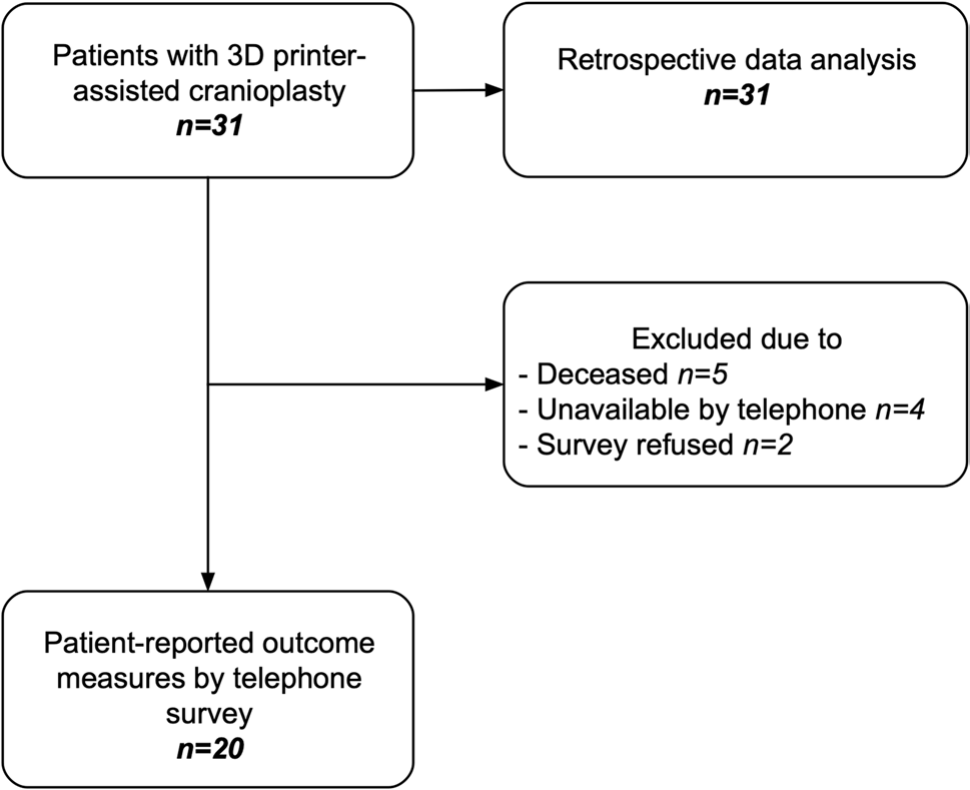
Flowchart displaying patients included for retrospective data analysis and the telephone interviews collecting the patient-reported outcome measures

We retrospectively analyzed data from 31 consecutive adult (≥ 18 years of age) patients who underwent patient-specific 3D printer-assisted cranioplasty at the Department of Neurosurgery at the University Hospital of Basel between 2012 and 2022. In addition, we assessed prospectively the patient-reported outcomes (PRO) of 20 (64.5%) patients using a telephone survey as a patient-reported outcome measurement (PROM). Five patients (16.1%) were deceased at the time of the telephone survey, five patients (16.1%) could not be reached by telephone after multiple attempts (> 4), and two patients (6.5%) declined to participate in the survey (Fig. 1). All virtual surgical planning and 3D printing procedures were performed in the point-of-care 3D print lab by the surgical team. The surgical procedures were performed in collaboration with the Department of Cranio-Maxillofacial Surgery at our institution. Patients with extensive bone defects, e.g., after hemicraniectomy or bifrontal craniectomy or with bone defects with difficult geometric configuration with high cosmetic and/or functional relevance such as in the frontotemporal region with or without orbital involvement, were considered for cranioplasty using the 3D printer-assisted cranioplasty technique. Adult patients undergoing in-house patient-specific 3D printer-assisted cranioplasty were included in this study. Patients with a documented refusal to participate in the research projects, where no patient-specific 3D printer-assisted cranioplasty planning was undertaken or where the preoperative planning was done in-house but an industrial company produced the implant, were excluded. Two of the four patients in whom the cranioplasty had to be removed due to infection could be interviewed by telephone. A new cranioplasty has already been performed in one patient, and a new cranioplasty is being planned for the second patient. In this case, the survey was based on the patient’s recollections just before the explantation of the cranioplasty.

**Fig. 2:**
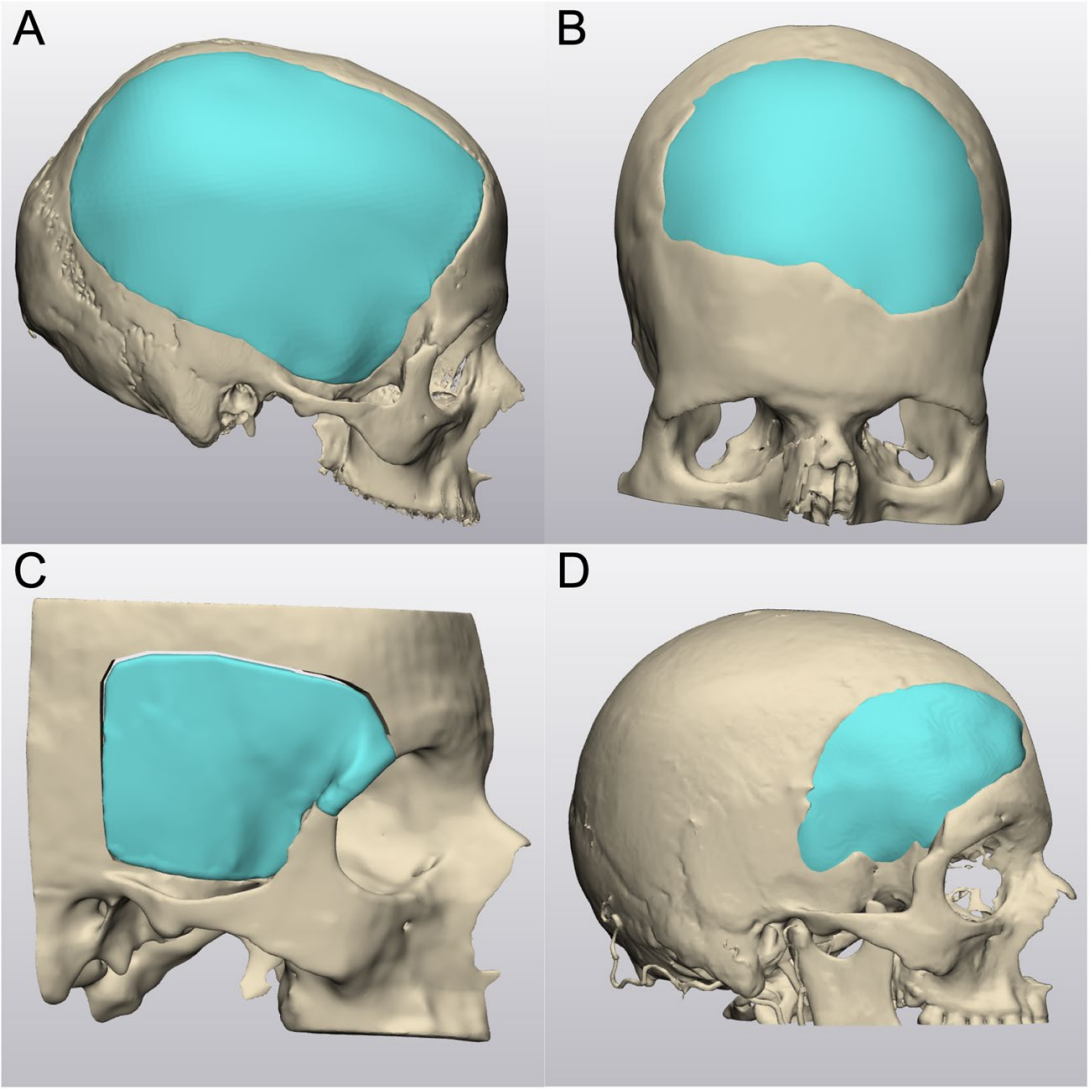
3D planning of a frontotemporoparietal (A), bifrontal (B), frontotemporal with orbital involvement (C), and a frontotemporal defect without orbital involvement(D)

Data were extracted from our surgical logbook and the patients’ medical files. We grouped the different localizations of cranioplasty into the following four categories: [1] frontotemporoparietal (e.g., after decompressive hemicraniectomy), [2] bifrontal, [3] frontotemporal without orbital involvement, and [4] frontotemporal with orbital involvement (Fig. 2).

The primary endpoint was the functional outcome measured by the modified Rankin scale (mRS) based on the patients’ medical files at discharge and the last follow-up. Secondary endpoints were Glasgow Coma Scale (GCS), surgical morbidity, length of hospitalization, mortality rate, and fabrication costs. Furthermore, we assessed PRO on cosmetic satisfaction using a 10-point scale (10 = very satisfied) and on further surgery and cosmetic-related factors based on a standardized survey (Supplementary Questionnaire). The PROM, which has not been validated so far, was developed in a multidisciplinary fashion by members of the Department of Neurosurgery (FE and JS) and the Department of Cranio-Maxillofacial Surgery (NS and FT) at the study site. The goal was to provide a quick, straightforward, informative assessment of patient assessment of cosmetics, palpable gaps around the implant, visible asymmetries of the head/face, and postoperative swelling and pain, and assess the mRS from the patient’s perspective.

Costs per implant were calculated based on costs for material (PMMA, polylactic acid (PLA), silicone), software licenses, hardware, personnel, and sterilization. An average hourly wage of 100 USD/h was assumed to calculate labor costs. The annual licensing cost for the software (18,000 USD) and the one-time acquisition cost for the hardware (5000 USD), which includes a computer workstation, a 3D printer, and any spare parts, were divided by the annual mean number of printed templates and models of 110 to calculate the cost per implant. In addition, a 5-year depreciation period was assumed for the hardware. Further, a subanalysis regarding the different localization categories was done for all outcome parameters.

This study, with its retrospective and prospective data collection and analysis, was approved by the local ethics committee (EKNZ, Basel, Switzerland), where patient consent was waived for the retrospective data analysis. Written patient consent was obtained for the telephone-based PROM.

### Patient-specific 3D printer-assisted cranioplasty technique

**Fig. 3:**
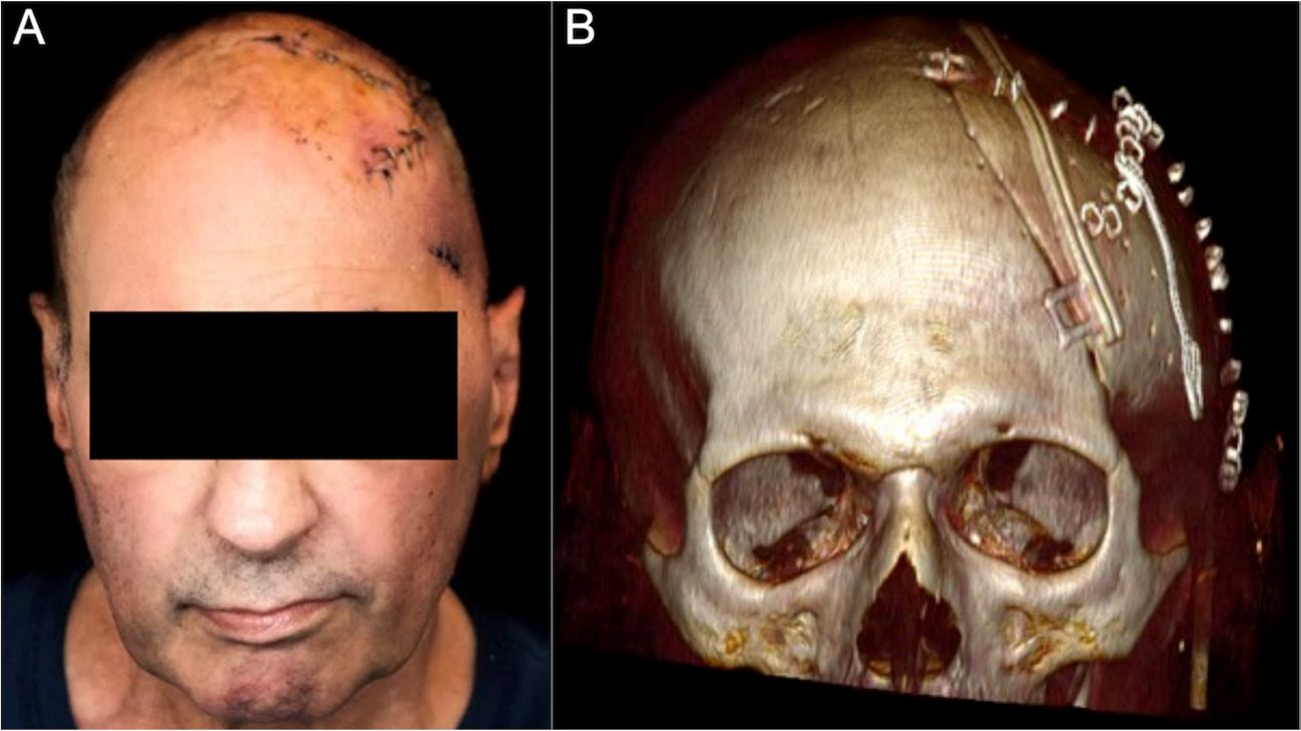
Censored photograph 1 week postoperatively after 3D printer-assisted cranioplasty of a frontotemporoparietal defect (A) and 3D reconstruction based on CT imaging showing an accurate symmetric cranial contour (B)

The technique of patient-specific 3D printer-assisted cranioplasty has been previously published and described by our group [2]. In summary, using 3D reconstruction software (Mimics Innovation Suite v. 20.0-21.0 and 3-matic v. 12.0-13.0; Materialise Inc., Leuven, Belgium), the PSI is planned based on the patients’ computed tomography (CT) scan and its bone structural defect (Fig. 2). In cases with temporal hollowing, the implant contours were remodeled during virtual planning to compensate for these soft tissue deficiencies. In patients with multiple scars and extensive soft tissue changes, we slightly under-contoured the implants in the virtual planning to enable tensionless wound closure. A PLA template is then printed using a desktop 3D printer (MakerBot Replicator+; MakerBot Industries, Brooklyn, NY, USA). Based on these templates, molds out of additive cross-linking silicone (Dublisil 30; Dreve Dentamid GmbH, Unna, Germany) are produced, sterilized in an autoclave in a certified and validated way (134–137 °C, over 18 min), and used intraoperatively to manufacture a PSI with PMMA (PALACOS R+G; Heraeus Kulzer GmbH, Hanau, Germany). Silicone has a low thermal conductivity and is therefore very heat resistant, which is why the material is well suited and autoclave temperatures have no effect on the silicone mold [5]. Before implant insertion, the implant was perforated at several points using a drill, allowing dural tack-up sutures to be placed whenever possible. In cases where the dura had to be resected and reconstructed, no or only single tack-up sutures were placed due to the fragility of the duraplasty. The implant was then fixed using titanium plates and screws (DePuy Synthes MatrixNEURO, Bettlach, Switzerland) or CranioFix (Aesculap AG, Tuttlingen, Germany). The temporalis muscle was dissected beforehand, pulled upward, and fixed to the bone using fixation sutures. A subgaleal drain was inserted in all frontotemporoparietal cranioplasties and removed on the second postoperative day (Fig. 3). In other cranioplasty locations, the surgeon decided individually, depending on the hemostasis, whether a subgaleal drain was inserted.

The regulation of the described in-house manufacturing process of patient-specific implants falls under an exception of Article 5(5) of Regulation (EU) 2017/745 (medical devices regulation, MDR) and Regulation (EU) 2017/746 (in vitro diagnostic medical devices regulation, IVDR). At present, according to this exemption, healthcare institutions must provide information on the manufacture and use of in-house devices to its competent authority upon request (Swiss Medic in Switzerland), which must include a justification of their manufacturing, modification, and use.

### Statistical analysis

All statistical analyses were done using SPSS (version 28.0, IBM Corp.). Univariate analysis was performed using the Fisher exact or chi-square test for categorical data and the Mann–Whitney U or the Kruskal–Wallis test for continuous data. A *p*-value < 0.05 was considered significant.

## Results

We included 31 consecutive adult patients (*n* = 17 male, 54.8%) with a mean age of 51.9 (± 16) years (range 22–90 years). The most common comorbidities were arterial hypertension (*n* = 10, 32.3%), epilepsy (*n* = 7, 22.6%), and cerebrovascular insult (*n* = 5, 16.1%). The most common preoperative symptoms were hemiparesis (45.2%), visual disturbance (12.9%), visible indentation around the bone defect (12.9%), and symptom constellations like matching sinking skin flap syndrome (9.7%). Seventeen patients (54.8%) had a preoperative mRS of between 0 and 2, and 27 patients (87.1%) had a GCS between 13 and 15 points (Table 1).

**Table 1:** Baseline and procedure characteristics of our case series

Patient characteristics	Number (percentage), *n* = 31
Demographic	
Mean age ± SD, years	51.9 ± 16
Males	17 (54.8)
mRS at baseline	
≤ 2	17 (54.8)
> 2	14 (45.2)
GCS at baseline	
13–15	27 (87.1)
9–12	2 (6.5)
3–8	2 (6.5)
Comorbidities	
Arterial hypertension	10 (32.3)
Epilepsy	7 (22.6)
Cerebrovascular insult	5 (16.1)
Cardiac arrhythmia	3 (9.7)
Coronary artery disease	2 (6.5)
Diabetes mellitus	1 (3.2)
Symptoms preoperatively	
Motor weakness	14 (45.2)
Visual disturbance	4 (12.9)
Visible and disturbing indentation around the bone defect	4 (12.9)
Symptom constellation for SSFS	3 (9.7)
Headache	2 (6.5)
Speech disturbance	2 (6.5)
Hypesthesia	1 (3.2)
Exophthalmos	1 (3.2)
Procedure characteristics	
Surgery duration (h) mean ± SD	3 ± 2.1
Blood loss (l) mean ± SD	0.4 ± 0.6
Location	
Frontotemporoparietal	19 (61.3)
Frontotemporal with orbital involvement	6 (19.4)
Frontotemporal w/o orbital involvement	4 (12.9)
Bifrontal	2 (6.5)
Indication for cranioplasty	
Infection of the previous bone flap/cranioplasty	14 (45.2)
Tumor affection of removed bone flap	6 (19.4)
Resorption of the previous bone flap	6 (19.4)
Disposal of the initial bone flap	4 (12.9)
Primary bone defect due to trauma	1 (3.2)

*SD*, standard deviation; *GCS*, Glasgow Coma Scale; *mRS*, modified Rankin score; l, liter; *w*, with; *w/o*, without

All values are presented as number (%) of patients or mean ± SD if not otherwise specified

The indication for patient-specific 3D printer-assisted cranioplasty was mainly due to prior infection (45.2%), unavailability due to tumor infiltration (19.4%), or due to resorption of the autologous bone flap (19.4%). Patient-specific 3D printer-assisted cranioplasty was used to reconstruct a frontotemporoparietal defect in 19 cases (61.3%), a frontotemporal with orbital involvement in six cases (19.4%), a frontotemporal defect without orbital involvement in four cases (12.9%), and a bifrontal defect in two cases (6.5%) (Table 1).

### Clinical outcome at discharge and last follow-up

**Fig. 4:**
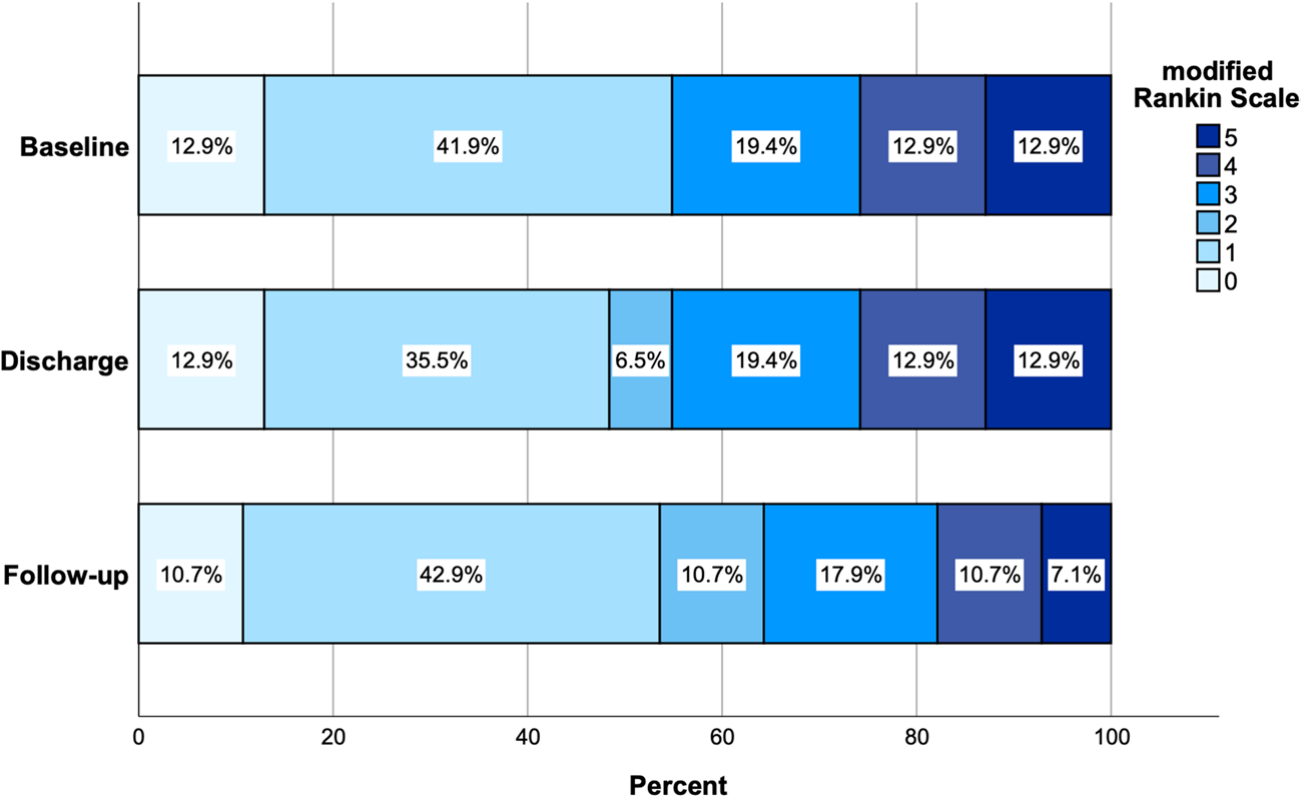
Stacked bar chart showing modified Rankin scale at baseline, discharge, and last follow-up

At discharge, 17 patients (54.8%), and at the last follow-up, 18 patients (58.1%) showed good clinical outcomes (mRS ≤ 2), which corresponds to an overall relative increase of 5.9% in the proportion of patients with a good clinical outcome at follow-up compared with admission (Fig. 4).

Clinical status at discharge showed improvement in 13 patients (41.9%), unchanged in 16 patients (51.6%), and worsened in two patients (6.5%) compared to admission. Two patients (6.5%) showed postoperatively new visual disturbances. In contrast, the GCS at discharge showed a slight improvement compared with admission, with 28 patients (90.3%) having a GCS between 13 and 15. The mean (range) time to discharge was 8.5 (4–25) days (Table 3).

At the last follow-up, 26 patients (83.9%) had a GCS between 13 and 15. The clinical status compared to discharge improved in 11 patients (35.5%) and was unchanged in 17 (54.8%). The 90-day mortality rate was 3.2%. One patient died 74 days after surgery due to refractory status epilepticus, most likely due to underlying parenchymal damage from aneurysmal subarachnoid hemorrhage with associated parenchymal hemorrhage. Four patients (12.9%) died after completion of follow-up unrelated to surgery at an average of 15 (± 11.7) months after surgery. The mean follow-up duration was 14.9 (± 19.8) months (Table 3).

### Patient-reported outcome

**Fig. 5:**
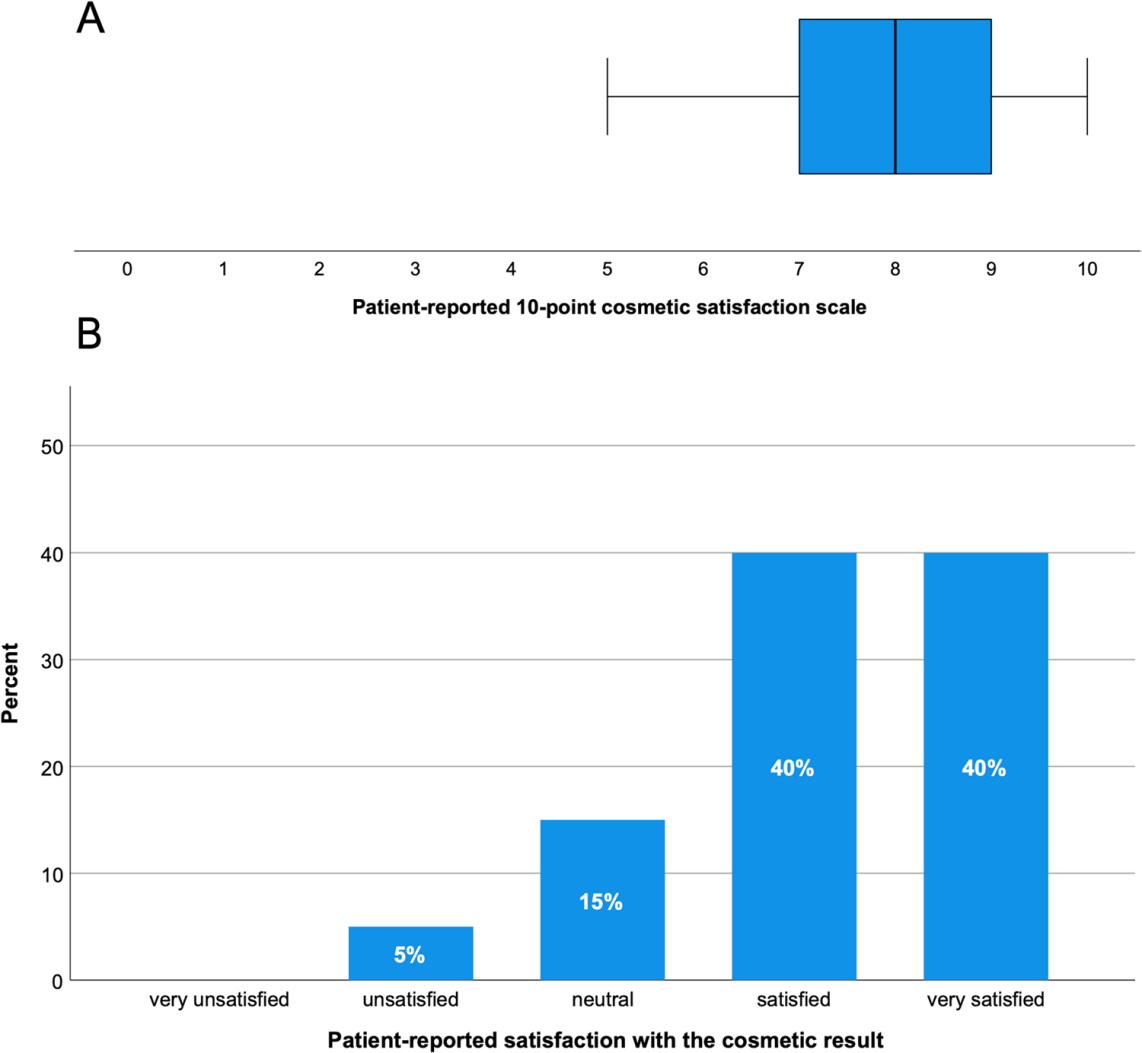
Boxplot representing the 10-point cosmetic satisfaction score (A) and a bar chart representing the different satisfaction groups (B)

The mean time from surgery to the telephone survey was 33.3 (± 20.6) months; 80% of the patients (*n* = 16) were satisfied or very satisfied with the cosmetic outcome. Out of the 20 patients (64.5%) who participated in the telephone survey, the average patient-reported satisfaction score regarding the cosmetic result was 7.8 (± 1.5) (Fig. 5, Table 2). One patient (5%) reported dissatisfaction with the cosmetic result because the implant protruded slightly beyond the bone margin at one site. However, the same patient gave a cosmetic satisfaction score of 6 points. Four (20%) patients reported noticeable gaps in the implant. Furthermore, seven patients (35%) reported visible asymmetry, seven reported transient postoperative swelling, and seven reported transient postoperative pain in the surgical area. Of the seven patients who described visible asymmetry, 85.7% (*n* = 6) were still very satisfied or satisfied with the cosmetic result with a patient-reported satisfaction score regarding the cosmetic result of 7.1 (± 1.3). In addition, we collected the current mRS by telephone, and 13 patients (65%) reported an mRS between 0 and 2 (Table 2).

**Table 2:** Patient-reported outcome measures

Patient-reported outcome measures	Number (percentage), *n* = 20
Time to survey postoperatively (m) mean ± SD	33.3 ± 20.6
Cosmetic satisfaction score mean ± SD (score 1–10)	7.8 ± 1.5
Cosmetic satisfaction, grouped	
Satisfied/very satisfied	16 (80)
Neutral	3 (15)
Unsatisfied/very unsatisfied	1 (5)
Palpable gaps around the implant = yes	4 (20)
Visible asymmetries = yes	7 (35)
Postoperative swelling = yes	7 (35)
Duration of postoperative swelling (d) mean ± SD	21.4 ± 20.6
Postoperative pain = yes	7 (35)
Duration of postoperative pain (d) mean ± SD	17.3 ± 19.9
Patient-reported mRS	
≤ 2	13 (65)
> 2	7 (35)

*m*, months; *d*, days; *SD*, standard deviation; *mRS*, modified Rankin score

All values are presented as number (%) of patients or mean ± SD if not otherwise specified

### Surgical details and complications

**Fig. 6:**
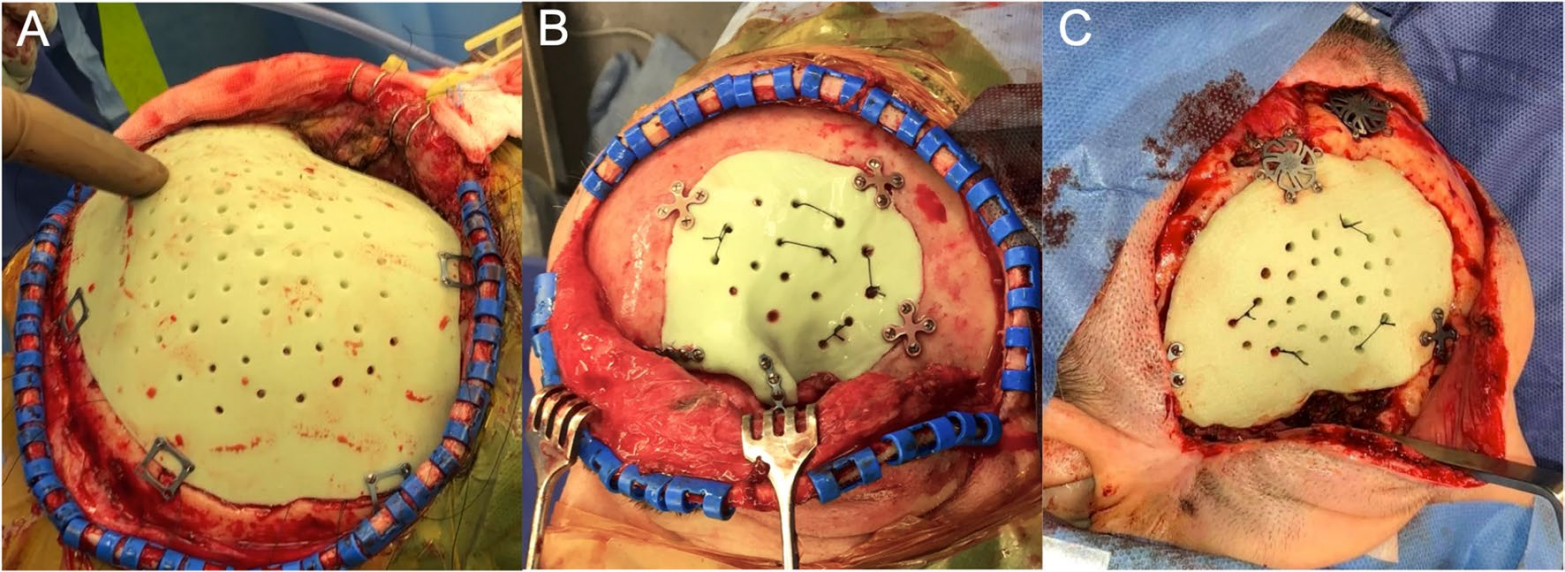
Intraoperative excellent accuracy of the patient-specific 3D printer-assisted reconstruction of a frontotemporoparietal (A), a frontotemporal defect with orbital involvement (B), and without orbital involvement (C)

The mean duration of surgery was 3 (± 2.1) h, and blood loss was 0.4 (± 0.6) L. All implants showed excellent intraoperative fitting accuracy (Fig. 6). Only discrete modifications had to be made with a high-speed drill. No intraoperative complications occurred in any of the 31 patients. Clinically relevant complications associated with surgery occurred in 11 patients (35.5%), of which postoperative epidural hematoma (EDH) occurred in four (12.9%), superficial infection in three (9.7%), deep infection in one (3.2%), new visual disorders in two (6.5%), and an unclear epidural fluid collection in one case (3.2%) (Table 3). 54.5% of the clinically relevant surgical complications, including the postoperative EDH (*n* = 4) and new visual disturbances (*n* = 2) occurred early (within 30 days postoperatively). Overall, ten patients (32.3%) underwent revision surgery, including four with EDH, four with infection, one with postoperative unilateral acute vision loss, and one patient who developed a very delayed (312 days postoperatively) progressive unclear epidural collection that was treated by epiduro-peritoneal shunt.

**Table 3:** Clinical outcome and surgery-related complications of our cohort

Outcome measures	Number (percentage), *n* = 31
Development of the clinical condition at discharge compared to admission	
Improved	13 (41.9)
Same	16 (51.6)
Worsened^*^	2 (6.5)
mRS at discharge	
≤ 2	17 (54.8)
> 2	14 (45.2)
GCS at discharge	
13–15	28 (90.3)
9–12	2 (6.5)
3–8	1 (3.2)
Development of the clinical condition at follow-up compared to discharge	
Improved	11 (35.5)
Same	17 (54.8)
Worsened	0
mRS at follow-up	
≤ 2	18 (58.1)
> 2	10 (32.3)
GCS at follow-up	
13–15	26 (83.9)
9–12	2 (6.5)
3–8	0
Surgery-related complications	13 (39.4)
Epidural hematoma/collection	5 (16.1)
Superficial infection	3 (9.7)
Deep infection	1 (3.2)
Postoperative new visual disturbances	2 (6.5)
Mild facial palsy	2 (6.5)
Revision surgery	10 (32.3)
Time to revision surgery (d) mean ± SD	179 ± 387
Length of hospital stay (d) mean ± SD	8.5± 5.2
Length of follow-up (m) mean ± SD	14.9±19.8
Mortality	
90-day mortality	1 (3.2)
Overall mortality^†^	5 (16.1)

*SD*, standard deviation; *GCS*, Glasgow Coma Scale; *mRS*, modified Rankin score; *d*, days; *m*, months

All values are presented as number (%) of patients or mean ± SD if not otherwise specified

*Two patients showed new visual impairment postoperatively

† No mortality was surgery related

One of the two patients with postoperative new visual disturbances developed postoperatively acute ipsilateral amaurosis and underwent emergency decompression of the optic nerve on the same day. The second patient showed transient slight double vision and was managed conservatively. The four patients with epidural hematomas received revision surgery within the first 4 postoperative days. The four patients with the infection received revision surgery with implant removal between 1.2 and 40.8 months postoperatively. So far, in one patient, a new cranioplasty combined with a latissimus dorsi myocutaneous flap has been performed after multiple wound revisions. In another patient, a new cranioplasty is now being planned after multiple wound revisions. In the remaining two patients, cranioplasty has not been scheduled yet. Furthermore, clinically irrelevant mild facial nerve palsy isolated to the frontal branch was observed during follow-up in two patients, which the patients neither noticed nor bothered. Permanent morbidity rate was 3.2% (*n* = 1). Unfortunately, the patient’s vision with postoperative secondary amaurosis did not recover despite the emergency revision surgery. The remaining patients recovered without permanent impairment.

### Costs

The mean manufacturing costs of a patient-specific 3D printer-assisted implant ranged from 748 to 1129 USD depending on complexity and size. Labor (or personnel) costs were the most significant drivers, accounting for 55 to 63% of total costs. The total time required to produce one implant was 225–405 min, corresponding to 416 and 716 USD labor costs, respectively. The mentioned production time extends over 3–4 days. The second largest cost driver is the hospital software license used for segmentation and design, which costs 164 USD (15–22%) per implant. Material costs for the PLA, silicone, and PMMA represent the third largest cost driver, averaging 110 to 190 USD (15–17%) per implant. The sterilization cost of the mold is 50 USD (4–7%) per implant. Finally, hardware costs were 9 USD (1–2%) per implant.

### Subanalysis of cosmetic and clinical outcomes regarding different defect locations

The PRO of cosmetic outcome did not show a significant difference between the four localization groups. In patients who underwent patient-specific 3D printer-assisted cranioplasty for a frontotemporal defect with orbital involvement, postoperative swelling tended to occur more often (80%) compared to the other sites. Furthermore, 68.4% (*n* = 13) of patients after cranioplasty for a frontotemporoparietal defect had an mRS of > 2 at discharge (*p* = 0.011). The surgery-related complications differed between the localization groups (*p* = 0.011); 21.1% (*n* = 4) of patients after frontotemporoparietal cranioplasty developed an EDH, which had to be evacuated by revision surgery. A new visual impairment occurred postoperatively in 33.3% (*n* = 2) of patients after frontotemporal cranioplasty with orbital involvement. The surgical duration of 3D printer-assisted cranioplasties for frontotemporal defects with orbital involvement took an average of 6.4 (± 1.9) h, which was significantly longer than the cranioplasty for the other defect localizations (Supplementary Table 1).

## Discussion

In our case series of 31consecutive patients undergoing patient-specific 3D printer-assisted cranioplasty, from an initial 54.8% of patients at baseline, 58.1% showed an mRS ≤ 2 during the last follow-up. In addition, clinical symptoms improved in 41.9 and 35.5% of patients at discharge and during the last follow-up, respectively. The mean cosmetic satisfaction score based on PROMs was 7.8 (± 1.5), with a satisfaction rate of 80%. The two most common defect locations were frontotemporoparietal (*n* = 19, 61.3%) and frontotemporal with orbital involvement (*n* = 6, 19.4%). One patient (3.2%) experienced permanent morbidity with complete ipsilateral vision loss postoperatively. The 90-day mortality rate was 3.2% (*n* = 1), with no surgery-related mortality.

### Clinical outcome and complications

Similarly, to literature reports on improved motor and neuropsychological function after cranioplasty, our series showed an improvement in clinical symptoms at discharge in 41.9% of the patients and a GCS improvement of 3% [6–8]. An increment of cerebral blood flow seems to be the most accepted hypothesis for clinical improvement after cranioplasty [9].

Most commonly, the literature focuses on the complications of cranioplasty of frontotemporoparietal defects after decompressive hemicraniectomy [10–14]. In a series of 166 patients, 85.3% received titanium cranioplasty, and 40.4% developed at least one complication. Infection was the most common (21.7%) [14]. A meta-analysis, which analyzed 3126 frontotemporoparietal cranioplasties, reported a mean complication rate of 19.5%, ranging from 3.9 to 45.3% [10]. In a study by Giese et al., a major complication rate of 21.7% requiring revision surgery was reported after they received an industrially manufactured patient-specific bone implant made from PMMA [13]. Similarly, our series shows a high rate of clinically relevant surgery-related complications (35.5%). This high complication rate is probably not related to or caused by the cranioplasty technique itself but rather due to the complexity and comorbidities of these patients.

Overall, in our series, 32.3% of patients underwent revision surgery due to a complication, most commonly an epidural hematoma/collection occurring in 16.1% of patients. Of these, two patients had a ventriculoperitoneal shunt, and one patient had known thrombocytopenia. These factors could have predisposed to the development of an epidural hematoma. This is comparable to published data where postoperative epidural hematoma, which had to be evacuated by revision surgery, occurred in 21.4% of patients [11]. The second most common complication in our case series was an infection in 12.9% of patients. This is consistent with the literature, which reports an infection rate of 10–26% after cranioplasty [15–17]. Epidural hematoma/collections and infections were most common in patients with frontotemporoparietal cranioplasty (36.9%). Among other factors, most probably the large wound area in frontotemporoparietal defects may favor the development of a postoperative epidural hematoma. In addition, all patients who underwent frontotemporoparietal cranioplasty had at least one previous operation at this site. This, in turn, leads to scar tissue with associated reduced tissue perfusion. Furthermore, in 45.2% of patients, the initial bone flap/cranioplasty was removed due to infection. This means that the same site was operated on at least twice in the case of an initial craniotomy, or three times in the case of an initial craniectomy. The number of previous surgeries is a known risk factor for surgical site infections and therefore might have contributed to the high infection rate in our study [18]. Routinely, we expose the dura or neodura and place the implant into the bony defect. A study by Gordon et al. reports a technique where a vascularized pericranium flap is left on the dura and the implant is placed on it into the bony defect, providing a “vascularized sandwich” encasing and protecting the implant [19]. With this technique, the authors report a low infection rate of 2.2%. However, the authors excluded smokers and patients with wound healing disorders; therefore, their infection rate is not comparable to ours. Reducing the complication rate in patients undergoing cranioplasty should be the focus of future interdisciplinary protocols and studies.

**Fig. 7:**
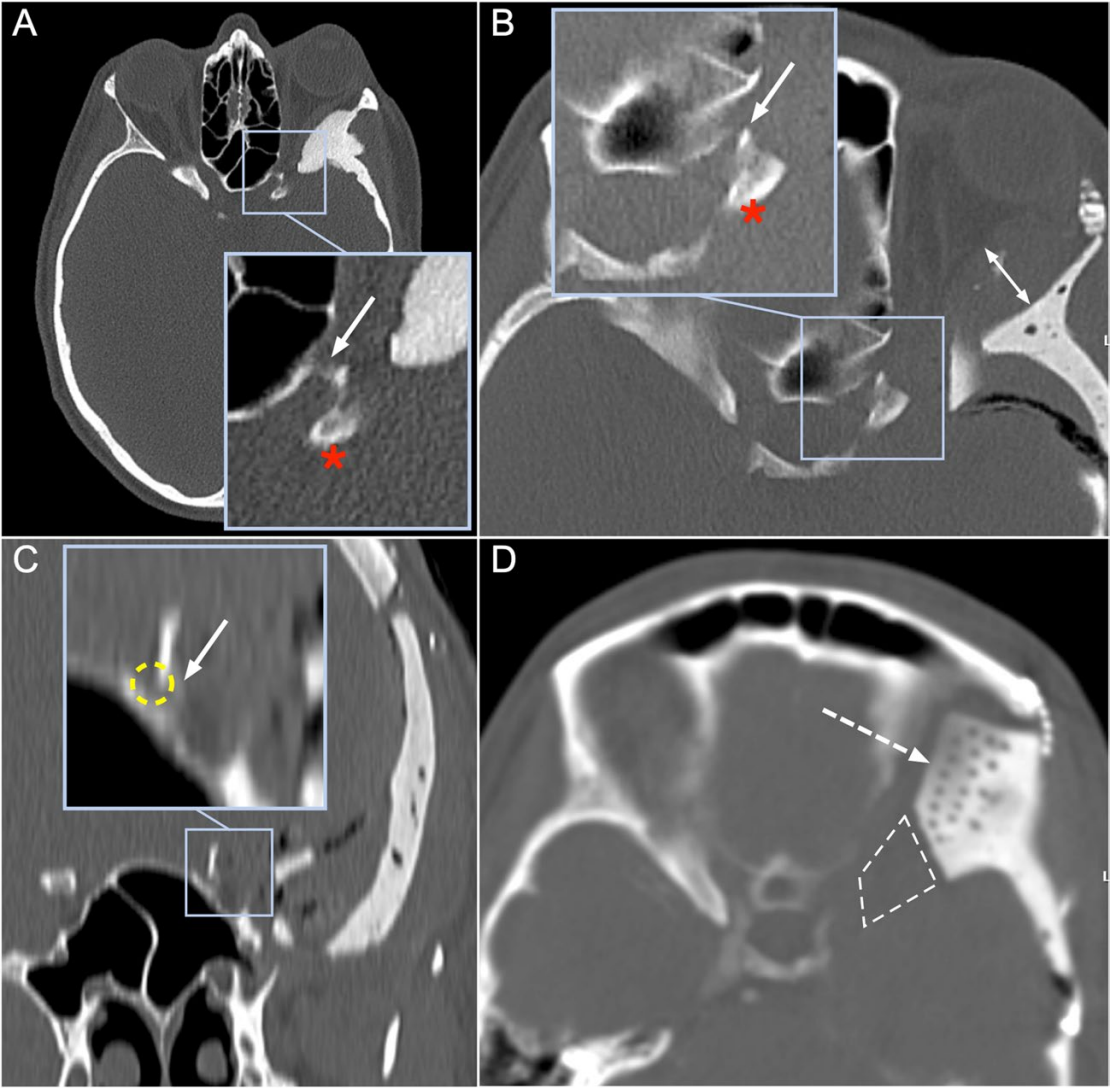
Preoperative axial CT showing the intraosseous meningioma of the lateral orbital wall, the optic strut (arrow), and the anterior clinoid (asterisk) (A). Postoperative axial CT after tumor resection and osseous reconstruction with a fractured optic strut (arrow) and dislocated anterior clinoid (asterisk), as well as intraorbital hematoma (double arrow) (B). Postoperative coronal CT shows a fractured optic strut (arrow) with potential optic nerve compression (yellow circle) (C). Axial CT after emergency revision and decompression in the region of the orbital apex (dashed square) and anterior clinoidectomy. Holes are seen in the implant (dashed arrow) to allow drainage of an intraorbital hematoma (D)

Two patients (6.1%) of our series who underwent a frontotemporal cranioplasty with orbital involvement developed postoperative new visual disturbances. These two complications were most likely directly associated with the patient-specific 3D printer-assisted cranioplasty technique. Cranioplasties involving the orbit are very demanding due to the complex geometry and carry the risk of compression of the orbital structures and can even lead to an orbital compartment syndrome in some cases [20, 21]. In the patient with complete vision loss, postoperative CT showed a dislocation of the anterior clinoid due to a fractured optic strut (Fig. 7B, C) and a lateral intraorbital hematoma (Fig. 7B) as possible etiologies. The dislocation of the anterior clinoid may have led to compression of the optic nerve, which was not noticed intraoperatively. Furthermore, the intraorbital hematoma probably increases intraorbital pressure and promotes vision loss. In the second patient with postoperative transient double vision, postoperative CT imaging revealed an intraorbital titanium mesh protruding slightly from the bone, which may also have resulted in intraorbital pressure elevation, causing the transient visual disturbance.

Therefore, reconstruction of the orbit must be performed very carefully, and a meticulous inspection and evaluation of the orbital pressure should be performed regularly. Furthermore, holes can be drilled into the implant to allow intraorbital blood drainage (Fig. 7D). Weighing optimal osseous reconstruction and perfect cosmetic results against the risk of compression of eloquent structures must be done carefully. Especially toward the orbital apex, we do not recommend a complete osseous reconstruction to reduce the risk of nerve compression even on account of less good cosmetic results.

### Patient-reported outcome

The cosmetic result after performing cranioplasty may substantially impact patients’ social life and self-esteem. Nevertheless, data on the cosmetic outcome after cranioplasty are scarce. Moreover, most studies that analyzed the cosmetic outcome did not consider the patient’s subjective opinion regarding the cosmetic result [4]. In a study by Fischer et al., who surveyed 46 patients undergoing freehand cranioplasty and in which the PMMA was shaped by hand without using a template or mold, an unsatisfactory cosmetic outcome was seen in 17.4% (*n* = 8). Accordingly, four patients (8.7%) requested revision surgery [22].

In contrast, our study revealed a dissatisfaction rate of only 5% (*n* = 1). On the other hand, satisfactory or very satisfactory cosmetic results were reported in 80% of patients. The mean cosmetic10-point satisfaction scale was 7.8 + 1.5, and none of the patients requested revision surgery. These results are also consistent with the literature, where excellent cosmetic outcomes were achieved using patient-specific 3D printer-assisted cranioplasty in approximately 88% of patients [23, 24].

Palpable gaps and visible asymmetry were reported by 20% (*n* = 4) and 35% (*n* = 7) of patients, respectively (Table 2). Almost all of these patients received 3D printer-assisted cranioplasty of a frontotemporoparietal defect after hemicraniectomy (Supplementary Table 1). These defects are large, and the patients have already undergone multiple previous surgeries. Probably, these reports of gaps and asymmetries are mainly due to soft tissue changes, such as atrophy of the temporal muscle or scar retractions. Since the planning of the implant mold is based on the bone defect in the CT, it is presumable that we achieved a very accurate bony reconstruction intraoperatively in all cases. As described above, if temporal hollowing is evident, the contour was adapted in the virtual planning to compensate for soft tissue deficiencies. Intraoperatively, the temporal muscle is additionally dissected and elevated with sutures. However, despite these measurements, often, with time, a retraction of the soft tissue occurs, leading to asymmetries perceived as disruptive by some patients.

To the best of our knowledge, our study is the largest case series reporting on the cosmetic outcome, where the patient-specific 3D printer-assisted cranioplasty technique was also used to reconstruct osseous defects involving the orbit. In our study, no differences in cosmetic outcomes between the different defect locations were found. Even in the frontotemporal cranioplasties involving the orbit, which were demanding due to the complex geometry, a cosmetic satisfaction score of 7.6 + 1.5 could be achieved (Supplementary Table 1).

It seems, therefore, that the patient-specific 3D printer-assisted cranioplasty technique described by us might be superior to the freehand technique regarding the cosmetic result. Especially for large frontotemporoparietal defects or defects involving the orbit, which have complex geometry, we were able to show that with the 3D printer-assisted technique, and a satisfactory cosmetic result could be achieved in most cases. Furthermore, a recently published study supports these findings, where 3D printed shells were used to manufacture the PSIs and achieve significantly better cosmetic results than the freehand technique, according to a composite cosmetic score, based on patient- and physician-reported assessment [25]. However, no cranioplasties with orbital involvement were performed in the mentioned study.

### Costs

Our study’s mean cost per implant was 748–1129 USD depending on the implant size and complexity.

Based on the method of cost calculation, the costs for 3D printer-assisted cranioplasty mentioned in the literature vary between 260 and 2780 USD [ 11,25,26]. One of the main reasons for the low cost calculation in the literature is that labor and licensing costs are often not considered.

A freehand cranioplasty costs approximately 55 USD [25]. However, possible secondary costs from revision surgeries due to inadequate cosmesis are not considered. The costs for autologous bone flap consist mainly of the cost of cryoconservation of approximately 600 USD, while costs for potential revision surgery in case of bone resorption were not considered [11]. Industrially manufactured PSIs made of titanium, PEEK, cost approximately 5000–10,000 USD [12, 25].

Virtual surgical planning and 3D printer-assisted fabrication of patient-specific PMMA implants at the point of care can be a time-saving, economical solution compared to industrial, externally manufactured customized implants. Various factors were considered to calculate the total manufacturing cost of patient-specific PMMA implants. There are significant scaling effects, i.e., with an increase in the number of implants produced per year and the use of existing hardware and software for 3D models (as in most 3D print labs), the manufacturing costs per unit decrease enormously. In these scaling effects, the license costs for certified medical segmentation and design software and the hardware costs play a major role. Labor costs depend mainly on which medical professional designs and manufactures the implant templates and the different country-dependent wage levels.

Overall, with the described 3D printer-assisted technique, comparable PSIs can be manufactured as the industrially manufactured PSIs but at 6–9 times lower costs.

## Limitations

These retrospectively collected data are subject to all the limitations inherent in such study designs. Unfortunately, not all 31 patients were available for telephone interview, and therefore the PRO are only represented by a part of the patients, which reduces the power of the reported data. Furthermore, validated scores and questions measuring cosmetics after cranioplasty do not exist to our knowledge, potentially limiting the significance of our PRO. In addition, the meaning of a satisfying cosmetic result was not defined. Clearly, the term “good cosmetic outcome” is subjective and not uniformly looked at among patients, which potentially lowers the significance of the results. However, the cosmetic outcome remains a subjective parameter and is assessed within our study subjectively by the patients themselves, reflecting their own opinion of “good cosmetic outcome.” The manufacturing costs of the implants were estimated retrospectively for the whole series and were not calculated for each implant separately, which in turn may result in a potential inaccuracy. Furthermore, the costs are highly dependent on the wage levels and especially on the number of implants produced annually, which is why the cost calculation cannot be directly transferred to other hospitals.

## Conclusion

Based on our results, patient-specific 3D printer-assisted cranioplasty leads to clinical improvement, with 58.1% of patients showing an mRS of ≤ 2 during follow-up; 80% of patients reported satisfactory cosmetic results even in geometrically complex cranioplasties such as frontotemporoparietal cranioplasties or frontotemporal cranioplasties with orbital involvement. This technique is a cost-effective alternative to expensive industrially manufactured PSIs. In the future, implants will be manufactured directly by 3D printing at the point-of-care, bringing innovative, patient-specific treatment options to many areas of surgery.

### Supplementary information

Supplementary file 1 **Supplementary Table 1.** Subanalysis of primary and secondary outcome measures regarding the different defect localizations

Supplementary file 2 **Supplementary Questionnaire**

## Abbreviations

3D: Three-dimensional
PMMA: Polymethyl-methacrylate
PSI: Patient-specific implant
PEEK: Polyetheretherketone
PROM: Patient-reported outcome measures
PRO: Patient-reported outcome
GCS: Glasgow Coma Scale
mRS: Modified Rankin scale
PLA: Polylactic acid
EDH: Epidural hematoma
