# Supplementary material for: Clinical and patient-reported outcome after patient-specific 3D printer-assisted cranioplasty
Source: Neurosurg Rev. 2023 Apr 19;46(1):93. doi: 10.1007/s10143-023-02000-9 (PMC10115682; doi:10.1007/s10143-023-02000-9)
Supplement: Supplementary file 1 — Supplementary Table 1. Subanalysis of primary and secondary outcome measures regarding the different defect localizations [file 10143_2023_2000_MOESM1_ESM.docx]

**Supplementary Table 1.** Subanalysis of primary and secondary outcome measures regarding the different defect localizations.

| Outcome measures | Frontotemporoparietal | Frontotemporal w orbital involv. | Frontotemporal w/o orbital involv. | Bifrontal | P value |
| --- | --- | --- | --- | --- | --- |
| **Patient-reported outcome measures** |  |  |  |  |  |
| No. of surveyed patients | 11 | 5 | 3 | 1 |  |
| Cosmetic satisfaction score mean ± SD (score 1-10) | 7.7 ± 1.6 | 7.6 ± 1.5 | 8.3 ± 2.1 | 8 | 0.886 |
| Cosmetic satisfaction, grouped |  |  |  |  | 0.386 |
| Satisfied/very satisfied | 9 (81.8) | 4 (80) | 2 (66.7) | 1 (100) |  |
| Neutral | 2 (18.2) | 1 (20) |  |  |  |
| Unsatisfied/very unsatisfied |  |  | 1 (33.3) |  |  |
| Palpable gaps around the implant = yes | 3 (27.3) |  | 1 (33.3) |  | 0.502 |
| Visible asymmetries = yes | 5 (45.5) |  | 1 (33.3) | 1 (100) | 0.166 |
| Postoperative swelling = yes | 3 (27.3) | 4 (80) |  |  | 0.075 |
| Postoperative pain = yes | 3 (27.3) | 2 (40) | 1 (33.3) | 1 (100) | 0.531 |
| Patient-reported mRS |  |  |  |  | 0.213 |
| ≤ 2 | 5 (45.5) | 4 (80) | 3 (100) | 1 (100) |  |
| > 2 | 6 (54.5) | 1 (20) |  |  |  |
| **Outcome measures out of retrospective data analysis** |  |  |  |  |  |
| No. of total patients | 19 | 6 | 4 | 2 |  |
| **mRS at discharge** |  |  |  |  | **0.011** |
| ≤ 2 | 6 (31.6) | 5 (83.3) | 4 (100) | 2 (100) |  |
| > 2 | 13 (68.4) | 1 (16.7) |  |  |  |
| **GCS at discharge** |  |  |  |  | 0.910 |
| 13-15 | 16 (84.2) | 6 (100) | 4 (100) | 2 (100) |  |
| 9-12 | 2 (10.5) |  |  |  |  |
| 3-8 | 1 (5.3) |  |  |  |  |
| **mRS at follow-up** |  |  |  |  | 0.065 |
| ≤ 2 | 7 (43.8) | 5 (83.3) | 4 (100) | 2 (100) |  |
| > 2 | 9 (56.3) | 1 (16.7) |  |  |  |
| **GCS at follow-up** |  |  |  |  | 0.659 |
| 13-15 | 14 (87.5) | 6 (100) | 4 (100) | 2 (100) |  |
| 9-12 | 2 (12.5) |  |  |  |  |
| 3-8 |  |  |  |  |  |
| **Surgery-related complications** |  |  |  |  | **0.011** |
| Epidural hematoma/collection | 4 (21.1) |  |  | 1 (50) |  |
| Superficial infection | 3 (15.8) |  |  |  |  |
| Deep infection |  |  |  | 1 (50) |  |
| Postoperative new visual disturbances |  | 2 (33.3) |  |  |  |
| Mild facial palsy | 1 (5.3) | 1 (16.7) |  |  |  |
| Revision surgery | 7 (36.8) | 1 (16.7) |  | 2 (100) | 0.116 |
| Time to revision surgery (d) mean ± SD | 33.7 ± 54.4 | 1 |  | 777 ± 658 | 0.062 |
| Length of hospital stay (d) mean ± SD | 8.6 ± 5.4 | 8 ± 1.3 | 5.5 ± 1.7 | 23 | 0.135 |
| Length of follow-up (d) mean ± SD | 421 ± 554 | 108 ± 98.5 | 862 ± 920 | 942 ± 866 | 0.18 |
| **Procedure characteristics** |  |  |  |  |  |
| Surgery duration (hours) mean ± SD | 2 ± 0.56 | 6.4 ± 1.9 | 1.9 ± 0.6 | 3.4 | **0.004** |
| Blood loss (l) mean ± SD | 0.28 ± 0.21 | 0.57 ± 0.48 | 0.16 ± 0.1 | 1.7 ± 2.1 | 0.185 |
| **Mortality** |  |  |  |  |  |
| 90-day mortality | 1 (5.3) |  |  |  | 0.859 |

SD = standard deviation, GCS = Glasgow come scale, mRS = modified Rankin Score, w = with, w/o = without, inolv. = involvement, d = days, m = months, l = liter.

All values presented as number (%) of patients or mean ± SD, if not otherwise specified.

*Two patients showed new visual impairment postoperatively.
