# Supplementary material for: Clinical and patient-reported outcome after patient-specific 3D printer-assisted cranioplasty
Source: Neurosurg Rev. 2023 Apr 19;46(1):93. doi: 10.1007/s10143-023-02000-9 (PMC10115682; doi:10.1007/s10143-023-02000-9)
Supplement: Supplementary file 2 — Supplementary Questionnaire [file 10143_2023_2000_MOESM2_ESM.docx]

**Supplementary Questionnaire**

**Patient-reported outcome measures (PROMs) for 3D printer-assisted cranioplasty**

1. Satisfaction with the cosmetic result
   1. Very dissatisfied
   2. Dissatisfied
   3. Neutral
   4. Satisfied
   5. Very satisfied
2. How satisfied are you with the cosmetic result
   1. Scale 1-10 (10=very satisfied)
3. Are gaps noticeable around the implant?
   1. Yes
   2. No
4. Are there visible contour irregularities or asymmetries?
   1. Yes
   2. No
5. Did you have any swelling after the operation?
   1. Yes
      1. How long? (days)
   2. No
6. Did you have pain after the operation?
   1. Yes
      1. How long? (days)
   2. No
7. According to the Modified Rankin Scale (mRS), how pronounced is your impairment in everyday life?
   1. 0 (No symptoms)
   2. 1 (no relevant impairment. Can perform daily activities despite some symptoms).
   3. 2 (Slight impairment. Can care for him/herself without help, but is restricted in everyday life.)
   4. 3 (Moderately severe impairment. Needs help in everyday life but can walk without assistance.)
   5. 4 (Higher degree of impairment. Needs help with personal hygiene, cannot walk without aid.)
   6. 5 (Severe disability. Bedridden, incontinent, requires constant nursing assistance.)
8. Further comments (as an open question)?
